# A Functional Contextualist Approach to Mastery Learning in Vocational Education and Training

**DOI:** 10.3389/fpsyg.2020.01479

**Published:** 2020-06-30

**Authors:** Daniel A. Parker, Elizabeth A. Roumell

**Affiliations:** ^1^Rehabilitation, Human Resources, and Communication Disorders, University of Arkansas, Fayetteville, AR, United States; ^2^Educational Administration and Human Resource Development, Texas A&M University, College Station, TX, United States

**Keywords:** VET, CVET, learning theory, mastery learning, deliberate practice, human capabilities, learning transfer

## Abstract

Along with technological progress, vocational education and training (VET) is consistently changing. Workforce disruption has serious consequences for workers and international economies, often requiring adults to transition into different occupations or to upskill to maintain employment. We review recent literature covering VET trends, theoretical considerations for the 21st century, and present an approach to workforce training to help workers not only learn necessary skills but also become adaptable to constant change. We suggest a functional contextualist approach to mastery learning achieves this aim. Specifically, we offer suggestions for pedagogy that not only develop skills but also encourage higher order thinking. Within a novice to expert continuum, we suggest deliberate practice, mental simulation, and reflective meaning making as methods to achieve efficiency and transfer—learning outcomes relevant to a changing workforce. This approach recognizes that learning is context bound and should promote broader human capabilities that support both employability and the continuing development of life literacies.

## Introduction

The flow of just about everything in the world—people, information, and things—continues to increase at an exponential rate. This is the 21st century norm. In an era of “Industry 4.0” (Schwab, 2016) and the “new collar workforce” (Rometty, 2016), many reports continue to point to broader national shortcomings across several areas of academic competencies (Carnevale et al., 2013) and in workforce skills (Organization for Economic Co-Operation and Development [OECD], 2017). Reports declare the necessity of upskilling national workforces (Ananiadou and Claro, 2009; Dunn, 2013; National Skills Coalition, 2013; Frey and Osborne, 2017) as well as the need for better-rounded life literacies and “soft skills” (U.S. Department of Labor [DOL], n.d.). To meet the requirements of a rapidly changing, competitive labor economy, workforce dynamics demand workers continuously develop new skills.

As technological progress increasingly disrupts the workplace, the modern worker is burdened with change and uncertainty. Depending on a society’s political economy, these changes can be devastating. Automation forces employees to either upskill—learn a new relevant workplace skill that is often more advanced than prior learned skills—or face layoffs. Sometimes, both are inevitable, leaving workers unemployed and forcing them to make difficult career decisions. In the United States, economists suggest workplace technological innovations, coupled with weak labor unions, are leading to labor polarization—stagnating wage growth between low and high paying jobs (Autor et al., 2006). While comprehensive, systemic solutions are required to address the big issues of labor rights and equitable wages, adult and workforce educators can presently ease worker anxiety by teaching skill adaptability within vocational education and training (VET). For many subject matter experts in VET, finding evidence-based practices for teaching adults is no easy feat. Roessger (2016) argues the adult learning literature essentially omits skills-based learning, favoring important, but often unrelatable approaches such as transformative and emancipatory learning. We hope to offer a more relevant approach through comprehensive mastery learning, extending mastery learning pedagogy beyond skills training and into reflective meaning-making.

Bloom (1968) and Keller (1968) popularized mastery learning in the mid-twentieth century with their approaches, “Learning for Mastery (LFM)” and the “Personalized System of Instruction (PSI),” respectively. Mastery learning claims that every learner can achieve mastery level status of a topic or domain given the appropriate amount of time and quality instruction. To achieve this aim, mastery learning proponents use instructional design methods that focus on personalized learning. The core procedures of mastery learning are (a) setting learning objectives for the domain or topic to be learned; (b) breaking the content into smaller units, each with their own set of objectives; (c) establishing a predetermined mastery-level criteria for learners to proceed from one learning unit to the next; and, (d) providing each learner opportunities for practice and feedback throughout the course (Block and Burns, 1976). Critics of mastery learning argue this approach is too mechanistic, rigid, or ineffective (Slavin, 1987); however, a meta-analysis suggests both LFM and the PSI techniques have positive learning outcomes, concluding a moderate effect size (SD = 0.52) on final exam scores compared to courses not using mastery learning (Kulik et al., 1990). Furthermore, low performing students tend to benefit most from mastery learning approaches (Ironsmith and Eppler, 2007). Recently, studies have focused on mastery learning in combination with simulation. Cook et al. (2013) found a large pooled effect size (*d*+ = 1.29, *K* = 41, *N* = 1,523) for mastery learning approaches in simulation-based medical education compared to other types of instruction.

With emphasis on demonstrating knowledge and skills, mastery learning is well suited to VET. For example, a trainee electrician must demonstrate fundamental knowledge of voltage, amperage, and resistance before proceeding to circuit design. Next, they must prove their ability to design circuits before moving on to more advanced tasks. When educators and trainers use standardized, procedural training, however, the learner’s context is often dismissed. Therefore, we advocate a functional contextualist approach to mastery learning in VET, using evidence-based practices to reach mastery while contextually situating the learner. Additionally, we focus on mastery learning techniques to achieve efficiency and learning transfer, valuable outcomes for workers who must adapt to a changing workplace environment. In this article, we frame mastery learning within the context of broader trends and practices in VET. Next, we discuss the functional contextualist approach to mastery learning and how it contributes to continuous VET. We then discuss the literature on efficiency and learning transfer as outcomes, and how mastery learning can achieve these outcomes through evidence-based skills learning and meaning-making practices. We conclude with implications for policy and directions for research.

## Current Trends and Practices in VET

In Tucker’s (2019) edited volume on international trends in VET, the authors assert that VET lives between national economic goal setting, economic development, national labor supply planning, primary and secondary education, tertiary education, business, and labor. Their work compares various national VET systems and grapples with the big ideas behind these systems and what they could mean in terms of educational and workforce development reform. They pose questions as to how we can work toward education and training systems that build a solid, yet flexible, academic and job skills foundation. Ideally, these systems would offer more equitable opportunities and empower adults in our societies to lead fulfilling lives with dignity—derived from doing work that is valued by others—and to participate in and contribute to their communities and civic society in meaningful ways.

Longstanding questions in the field of VET reflect some of the most pressing challenges related to education and vocational preparation in a rapidly changing economy. Some perspectives foreground technical and work-ready skills and competencies that emphasize a more instrumental approach to training and workforce development in order to address the current labor and skills gaps (Ananiadou and Claro, 2009; Voogt and Roblin, 2012). Other perspectives suggest that life-literacies, social and emotional intelligence, ethical decision making, autonomy, critical thinking, and the ability to collaborate, as emphasized in humanistic and more academically focused education, should take precedence in shaping healthy learning dispositions (East et al., 2014; National Centre for Vocational Education Research [NCVER], 2018). Furthermore, some take critical approaches to VET, criticizing capitalist motives to place profit over the welfare of the worker (Mannino and Faraci, 2017). For example, Suissa (2004) argues that integral education, combining both liberal and vocational education, provides a more equitable outcome for workers. Grounded in a social anarchist position, she cites capitalism as creating a false dichotomy between “mental” and “manual” work. Rather, education ought to focus on both perspectives, with the goal of abolishing this distinction and prioritizing freedom from oppression. Each of these preferences make valid arguments, and yet somehow still find agreement in that both educational forms (instrumental and humanistic) are necessary and ultimately complimentary. Views about what is to be included and which institutional forms deliver specific educational services in VET varies dramatically, and there is an increasing blurring of lines between general education and job-related training. Given these complexities and challenges, it is difficult to develop an authoritative definition for VET. For our purpose here, we understand VET as encompassing the variety of forms of learning that are primarily aimed at preparing for and supporting entry into the worlds-of-work, including initial entry, re-entry, and retooling (McGrath, 2012).

Interestingly, despite the recent renewal of interest in policy and VET programmatic matters, much of this debate and discourse does not center or address learning theory, pedagogical approaches, or best-practices in supporting learning that combines classroom-based academics and workplace-based training. The longstanding debate about how much general academic and humanistic education is necessary in complimenting developing job-related skills has not seemed to produce much contemporary theorizing related to VET teaching and learning (Hordern, 2014).

### VET for Development

Given the return of interest in VET as a mechanism for community and societal development (from a more international development perspective), which considers the purposes, natures, and possibilities of VET as a mechanism for human development, McGrath (2012) argues that it is time to return to theorizing VET learning. From this human development perspective, VET should entail a more inclusive view of education that goes beyond work and productivity and the orthodox view of VET for economic development (Choo, 2017). Rather, it should move toward the broader “vocation of being human.” Anderson (2009) asserts that the human condition is complex and interdependent, and the source of meaning of one’s occupation is inextricably linked to social relations, cultural understandings, and contextual aspects of their surrounding environment. People are not just potential workers, they are human beings, family members, and citizens with a wide range of needs, relationships, duties, interests, hopes, and goals—all of which go beyond their role as mere labor. Ideally, the learning theories from which we derive our VET educational models would reflect this context-bound nature of life, living, working, and learning (Robeyns, 2006; Choo, 2017).

Unfortunately, a focus on employability and human capital development poses serious limitations in both theoretical purchase and practical efficacy when it comes to skills development, learning, and creating meaning (Tucker, 2019). An overly instrumental skills focus is too shortsighted and may not cultivate much needed lifelong learning processes (Spring, 2015). Ultimately, common VET approaches remain underdeveloped in teaching and learning theory in both schooling and job-training contexts. Much of this is the result of outmoded VET educational models and stale theoretical paradigms. “As VET is about humans learning, working and living, it is imperative that it draws more consciously from a rich stream of theoretical insights” (McGrath, 2012, p. 625). The current performativity approach lacks interest in ethical questions concerning human flourishing and developing human capabilities, well-being and security, and matters of equity and social inclusion (Choo, 2017).

Derived from Sen (1999) and Nussbaum (2003, 2011), *human capabilities* are understood as *potential functionings* that include a wide variety of activities. These would include activities related to obtaining nourishment, finding shelter, access to healthy water and resources, being mobile, becoming educated, having fairly compensated and meaningful work, being safe, respected, maintaining individual and cultural dignity, and taking part in one’s family and community, etcetera (Walker, 2006). This perspective encompasses the notion of *life-literacies* which require lifelong and lifewide learning approaches (Baildon and Damico, 2011). The primary focus is developing peoples’ *agency freedom* and their ability to bring about change that is personally meaningful and of value. While a human capabilities perspective has grown in international education and development, this vision also has important implications for tertiary education and workforce development (Walker et al., 2010; Walker, 2012). But in order to pursue such educational aims, theoretical foundations in teaching and learning must also be revisited (Choo, 2016).

### From Learning Theory to Practice in VET

Vocational education and training is comprised of a large percentage of workplace learning (WPL), where learning takes place in and is shared between both classroom learning and job training in real companies or similar workplace scenarios and institutions. The contextualization of WPL is claimed to improve transfer from school to work (Tuomi-Gröhn and Engeström, 2003; Cameron et al., 2011) and student motivation (Kersh and Evans, 2010). The idea is to facilitate the connection between learning and practice, contribute to the development of work competencies, “learning to learn,” and professional dispositions. Unfortunately, research provides conflicting accounts on the effectiveness of common approaches to WPL (Islam, 2019). “Knowledge about how and to what extent students develop the intended competencies during WPL, how guidance takes place, and how theory and practice are attempted to be connected is insufficient” (Poortman et al., 2011, p. 268). Educational models and learning practices that have better theoretical grounding and explanatory purchase within VET are needed to gain more insight into these learning processes and their outcomes. Unfortunately, research with firm theoretical underpinnings that is also practically relevant to VET is limited (Hordern, 2014).

#### Traditional Learning Theory

Most learning theory, as taught in graduate programs for example, is presented as an individually oriented process of knowledge acquisition. This includes classic behaviorist perspectives, which focus on behavioral changes as evidence of learning, or cognitivist perspectives, which focus on the mental mechanisms for internalizing and processing information. These perspectives perceive learning as a change in behavior or information processing, suggesting learning is the product of inputs and outputs independent of the holistic learner. Many instructional design theories stem from these epistemological paradigms. Also common, following the interpretivist turn in social sciences, are constructivist perspectives of learning that focus more on the internalized meaning-making process that take place within the mind. From this perspective, knowledge is something that is unique to a person and is constructed through their personal experiences and contexts. Expanding on this notion, social constructivism presumes that all meaning making is social in nature and is created between people, taking social interaction into greater account. In this view, knowledge is something that is actively shared and generated between people as a social process (Merriam et al., 2007).

#### Integrative Approaches to Learning

Many perspectives and theories on learning have been derived from these established conceptualizations, and both paradigms have continued to produce additional theories and models of learning within education (see Illeris, 2018 for a useful overview of the history of learning theory). Some of these variations emphasize context and socialization, while others continue to refine individualistic processes of knowledge acquisition and skills development. Some theoretical variations, however, try to relate individual learning to social contexts (Illeris, 2003, 2004, 2007). For example, in VET, Poortman et al. (2011) propose a “comprehensive learning theory” that integrates cognitive, social, and affective dimensions and apply it to VET contexts. They assert, “The social and cognitive dimensions of learning should be united by combining the ‘acquisition metaphor’ and the ‘participation metaphor”’ (Poortman et al., 2011, p. 268), and that the relationships between individuals’ knowing and the social world are inseparable. Illeris’s theory first outlines various possible social and contextual interactions that can work as a stimulus for learning, which is then followed by a process of individual internalization of the content and meanings as a complimentary and interdependent process. Their approach, however, remains focused on the individual internalizing information, which runs the risk of reducing contextualized activity to ontogeny or biography.

#### Recontextualization

Hordern (2014) employs the notion of recontextualization to examine the process of how VET knowledge is developed, transformed, and transferred into practice. This approach is derived from sociological understandings of educational knowledge that examines the interrelation of distinct knowledge structures and the social dynamics of VET infrastructure. Recontextualization can be understood as the “socio-epistemic formation of vocational knowledge, as knowledge is assembled and recontextualized to meet the objectives of practice” (Hordern, 2014, p. 24). From this perspective, more generalized knowledge systems are adapted as they are applied across varying vocational contexts. The recontextualization of knowledge helps shed light on how knowledge is produced, validated, and made available to those in vocational practice. Barnett (2006) proposes that VET knowledge develops through a process of ‘*reclassificatory recontextualization*’ (Bernstein, 2000). This process integrates occupational and organizational problems with disciplinary knowledge to produce a ‘toolbox of applicable knowledge’ by restructuring disciplinary knowledge for vocational purposes and application. Barnett’s macro-level, sociological theoretical perspective sheds light on knowledge systems within VET and how they are adapted as applied, yet reveals little in terms of pedagogical VET practice.

#### Activity Theories

*Activity theories of learning* are learning theories based on the general “activity theory paradigm” a la Vygotsky and expanded on by Engeström et al. (2003). Engeström et al. (2003) convey, “Human activity is endlessly multifaceted, mobile, and rich in variations of content and form. It is perfectly understandable and probably necessary that the theory of activity should reflect that richness in mobility” (p. 20). The aim is to acknowledge that situated action moves us beyond mentalistic notions of individual information processing and past the dualism of imposed structure and individual experience (i.e., the structure/agency debate). An overly individualistic focus on learning poses challenges in accounting for socially distributed and collective aspects as well as the artifact-mediated or cultural aspects of human behavior.

From this view, an activity system is understood as a multilayered network of interconnected activity systems, and individual activities are understood as events within a collective activity system (Engeström et al., 2003). An “expansive cycle” of that activity system would entail both internalization of meaning and models from the activity system in which they are embedded, where those meanings are internalized. Subsequently, those models are then adapted through reflective appropriation and analysis and a new model is formed, implemented, and externalized (performed). This activity process of internalization, analysis, recreation, and externalization can be viewed as a form of iterative mastery learning where activity comes to be understood as students learning to use current models, which are then recreated and transcended. As students apply models in context, correcting for inconsistencies and contradictions in those models, they create new meaning and adapt the models. Activity theory, then, serves as a theoretical backdrop for both cyclical individual-level mastery learning and additional layers of systems learning (i.e., the above noted recontexualization theory, Hordern, 2014) that are constantly being tested and adapted as necessary. Activity theory integrates individual internalization of meaning as well as broader adaptive sociocultural and sociostructural meaning-making systems.

Like other integrated approaches, functional contextualism blends sociocultural and historical context, but with the pragmatic and scientific aims of behavior analysis. In addition to taught and constructed learning, functional contextualists suggest functional change in meaning is the process of recontextualization. Inspired by American pragmatism and Skinnerian behaviorism, functional contextualism takes a decidedly practical approach to instruction, suitable for a changing VET environment. Mastery learning particularly benefits from a functional contextualist philosophy, steering the technique away from a purely procedural strategy to one which encompasses contextualization and meaning making as core principles. In the following section, we describe functional contextualism and how it contributes to continuous VET.

## Functional Contextualism

All human activity is the lived performance of a learning philosophy. In its broadest sense, this entails human activity in individuals, groups, or societies, accounting for biological and environmental context. Reflexive learning is found throughout the animal kingdom, but derived learning—learning from constructing meaning—is uniquely human. This human behavior is what we desire to understand and explain. As noted previously, learning theories are derived from a multitude of philosophical approaches. However, with its emphasis on contextualization and learning behavior, we propose that functional contextualism—as defined by Biglan and Hayes (1996)—is best suited as a philosophical foundation for VET learning.

Functional contextualism is derived from one of Pepper’s (1942) “world hypotheses”—contextualism. Epistemologically, contextualists are pragmatists (Hayes et al., 1988), meaning they do not concern themselves with objective versus relative reality arguments. Contextualists know what they know “because it works,” and they have no interest in making claims about a universal reality. Contextualism is also distinct in what Pepper calls its “root metaphor.” Root metaphors are common sense analogies that capture the essence of a world hypothesis. For contextualism, the root metaphor is the “historical event,” also known as the *act-in-context* (Hayes et al., 1988). An act-in-context not only considers an act itself, but also the context in which the act exists. For example, if an industrial technician programs a Programmable Logic Controller (PLC), they are not only configuring software, entering code, typing on the keyboard, using the mouse, and sitting at a desk. Rather, they are doing *all* of it. Additionally, the industrial technician’s history and prior behavior all factor into the specified context. What skills did the technician obtain from formal education versus on-the-job training? Did they tinker with electronics as a child? What cultural factors influence their learning? Contextualists consider the *entire situated act* in their analysis (Fox, 2006).

Functional contextualism holds the same assumptions, but with analytic aims of prediction and influence (Hayes and Long, 2013). For example, clinical psychologists seek to determine what factors *predict* depression and what therapies work to *influence* the treatment of depression. VET researchers and practitioners may seek similar goals. Researchers might aim to *predict* factors that enhance critical thinking and create interventions that *influence* critical thinking for workers. In turn, vocational educators would employ pedagogies that consider these factors within practice. Influence, like other actions, is dependent on the act-in-context. Therefore, a functional contextualist researcher must seek to influence the context to influence the act (Hayes et al., 2001b). This does not mean altering the context itself. For example, if the learning context entails historical events, a practitioner cannot change the past or previous experiences. However, a practitioner can influence the function of the context; they can help learners alter the meaning of their context. If a learner developed a fear of failure from an historical event, a practitioner could aim to functionally change the meaning of that fear, perhaps through exposure therapy and confidence building.

For functional contextualists, prediction and influence are inseparable. Often, the words “prediction and influence” are used as one word to specify the integration of the two aims. Functional contextualists not only seek to predict events but also to influence changes in human behavior (Biglan and Hayes, 1996). Hayes et al. (2012) state it this way: “causal analysis in a functional contextual approach ultimately must extend to the manipulatable context of action” (p. 5). Therefore, researchers who can predict, but not identify factors which influence an outcome, have not yet discovered a “truth” in the pragmatic sense. This is not to say prediction or other types of research are not beneficial in a functional contextualist approach. Rather, other research informs hypotheses that lead to a prediction and influence aim. To achieve these aims, functional contextualists intentionally and systematically employ the scientific method. Hayes et al. (2012) write:

As a functional contextualist sees it, the ultimate purpose of behavioral science is to change the world in a positive and intentional way. Science is taken to be an empirical strategy of interacting in and with the world so as to learn how to be more effective in organizing it, speaking about it, measuring it, and changing it (p. 2).

Functional contextualists do not use the scientific method to discover universal truths, nor do they claim the scientific method is the only tool of inquiry. However, science is understood to be one of the more consistent and effective tools for discovering interventions that work to predict and influence behavior.

### Relational Frame Theory

Examining mastery learning and VET in purely procedural terms lends itself well to any behavioral theory. While functional contextualism underlies the procedural nature of skills-based learning, it further extends into higher level thinking and reflective meaning making. Like traditional behaviorism, functional contextualism rejects mechanistic perspectives of the mind. Unlike traditional behaviorism, however, functional contextualism does not dismiss thoughts—or the internal world of the mind—as irrelevant. This is evident in an influential theory derived from functional contextualism, *relational frame theory*. Relational frame theory (RFT) is a theory of verbal behavior and higher order cognition. While an exposition is well beyond the scope of this paper (see Hayes et al., 2001a; Törneke, 2010; Dymond and Roche, 2013), RFT posits higher order cognition is developed through verbal behavior. Verbal behavior, in turn, is composed of learned relational behavior (e.g., “I was taught football is a type of sport”) and derived relational behavior (e.g., “If sports are competitive, and football is a type of sport, football is competitive”).

Situated within functional contextualist philosophy, verbal behavior is also functionally dependent on context. Take, for example, the vocational occupation of welding technician. A person in the United States might hear “welding technician” and relate it to an undesirable occupation due to the cultural perception of welding as a career with low social value. However, upon learning the average salary of a welding technician, the context and meaning may change for that person. They may value the salary more than social perception, thus causing a functional change in meaning for “welding technician.” As we discuss in the next section, RFT has implications for contextualized higher order learning within a mastery learning approach.

## Mastery Learning and the Novice to Expert Continuum

Mastery learning assumes a developmental process of learning. Many theorists have also made this assumption in human learning. Piaget (1936) observed this in children, noting four distinct developmental stages. Piaget’s third and fourth stages are especially relevant, as they represent the transition from thinking in concrete terms to thinking abstractly. Taking a constructivist-developmentalist approach, Kegan (1982) further elaborates on Piaget’s theory of learning and development. Particularly, he emphasizes the social and environmental influences on a person’s meaning making processes. Within his five developmental stages, Kegan argues that people transition from viewing themselves as “subject” to “object.” That is, development becomes a process in which people differentiate their prior meaning of the world and assimilate new knowledge into their mental model. Like constructivists, functional contextualists perceive meaning making as a process of assimilating new knowledge into one’s prior experiences. However, functional contextualists also posit that assimilation is a *relational* and *functional* process that is context bound. For VET mastery learning, this means ensuring learners have competency in fundamental skills and leveraging the functional nature of their experiences to achieve improved learning outcomes. We argue this approach leads to improved learning efficiency and transfer, outcomes most suited for continuous learning in VET. Workers who can efficiently learn new skills and transfer learning to relevant contexts are better equipped to adapt to continuous change.

### Learning Fundamental Skills

Piaget (1974) argues that concrete learning is a prerequisite for abstract learning, especially for fields such as math and physics. Ausubel (1964) further argues that once students have foundational knowledge, they can make meaning by associating abstract concepts with both concrete knowledge and other abstract concepts. Several models and techniques exist to facilitate the fundamentals of skills-based mastery learning. Although more popular in childhood and special education, the *concrete-representation-abstract* (CRA) model—sometimes called the concrete-semiconcrete-abstract model—is a relevant strategy for VET. In CRA instruction, concrete subjects are learned before students progress to more abstract concepts. For example, in electronics, students would learn a concrete concept first, such as DC motor construction, then a representation of the concept, such as interpreting a DC motor design schematic, before continuing to abstract properties, such as electromagnetism. This way, students establish basic, familiar knowledge to relate to more difficult, abstract concepts. CRA techniques have made significant progress in mathematics education (Kamina and Iyer, 2009; Mudaly and Naidoo, 2015) and problem solving (Flores et al., 2016), particularly for students who struggle with learning abstract concepts.

**FIGURE 1:**
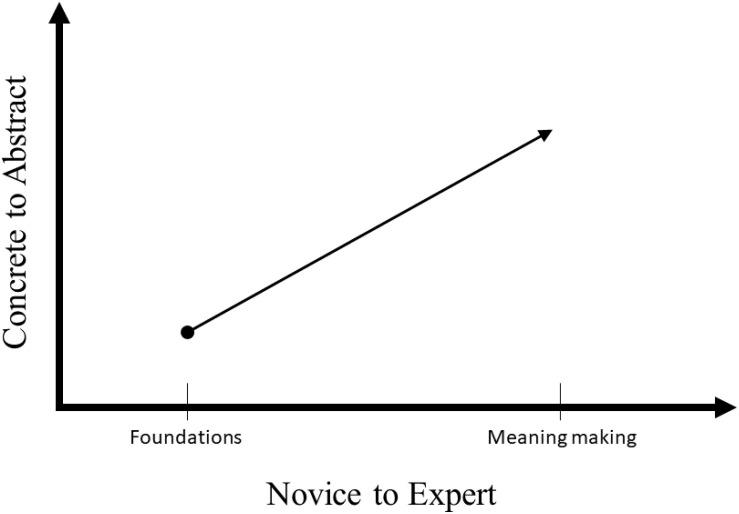
Mastery learning progression. This figure indicates the novice to expert continuum in a mastery learning approach.

Unlike Piaget, however, Ausubel argues against developmental stages, stating that meaning making occurs individually through both biological and environmental influences. Functional contextualists also take this approach to development, in which mastery learning is reinforced through personalized assessment and instruction. While CRA helps learners connect ideas to the physical world, it is improved by traditional methods of direct instruction common to mastery learning (Sealander et al., 2012). Roessger (2016) summarizes this research into a “novice to expert” framework. The novice to expert continuum, aligned with the concrete to abstract continuum, creates a framework for a functional contextualist approach to mastery learning (see Figure 1).

Stages of training and education must address both processes of knowledge acquisition and retention. Cognitive and behavioral science research demonstrates that direct instruction has significant positive effects on skills-based learning outcomes. Below we further explain some of the benefits of deliberate practice—such as retrieval practice, feedback, and distributed practice—as well as mental simulations and reflective meaning making.

### Deliberate Practice

The principle technique for foundational learning is practice. While this may be obvious, Roessger (2016) specifies practice must be deliberate, requiring feedback, variability, and focused incrementation. While achieving automaticity—unconsciously replicating similar performance—is beneficial for certain skills, learners must progress beyond automation to prevent stagnation in practice. Variability and focused incrementation in practice prevent automation. Roessger argues variability further increases adaptability in learners, supporting the generalization of skills and learning transfer. Additionally, focused incrementalism is not only vital to successful practice but is also a fundamental characteristic of mastery learning. By progressively increasing the difficulty of practice, learners avoid overly rigid automaticity ensuring continued learning and growth. Focused incrementalism also avoids stagnation by challenging learners in their understanding of the content. In mastery learning, this is achieved by obtaining a predefined mastery level in a content area before progressing to new material. Even so, finding the sweet spot of difficulty is a consistent challenge to educators. If practice becomes too difficult, the learner loses interest. However, if it is too easy, they are likely not learning anything. Using machine learning analysis, Wilson et al. (2019) found that about a 15% failure rate is optimal for progressive learning.

#### Retrieval Practice

*Retrieval practice* is the “act of calling information to mind rather than rereading it or hearing it” (Roediger and Butler, 2011, p. 20). The process of retrieval practice is simple. Students receive some form of instruction on a subject, and rather than repeatedly reading the material or taking notes, they attempt to recall the material. While retrieval practice focuses more on cognitive rather than psycho-motor skills, it is useful for efficiently learning technical terminology and procedures. By practicing recall, students learn the material better and are less prone to forgetting it (Roediger and Butler, 2011; Brown et al., 2014). Some researchers have investigated retrieval practice in training settings. For example, in an experiment with medical students, Kromann et al. (2009) found retrieval practice in a resuscitation training significantly improved outcomes in a simulated assessment of resuscitation skills.

#### Targeted Feedback

Like direct instruction methods, practice benefits from feedback and variability (Dunlosky et al., 2013). Consistent research demonstrates the importance of feedback in learning outcomes (Kluger and Denisi, 1996). Correct feedback is particularly important for recognition tests, such as multiple-choice examinations. As students learn from testing, a wrong answer without correct feedback may reinforce the wrong answer. Roessger (2016) further stresses the timeliness and duration of feedback, citing studies that demonstrate immediate feedback which occurs more often is most effective. In medical education, Snyder et al. (2011) found both human and virtual feedback were equally effective in a simulated mastery learning approach, indicating the positive effects of feedback regardless of delivery.

#### Distributed Practice

In most training situations, distributed practice, or variability, is necessary to mitigate automation and improve problem-solving. In the learning sciences literature, variability is split into two concepts: spacing and interleaving. *Spacing* is the optimal time for a learner to revisit the material, while *interleaving* is the method of interspersing material with other relevant material (Brown et al., 2014). Agarwal et al. (2017) notes these can be particularly important for learners with lower working memory capacity.

Researchers have consistently demonstrated that the repetition of study sessions with temporal gaps improve retention more than “massed” or block studying (Cepeda et al., 2009). Spacing essentially emphasizes the power of repeated practice. In the distributed practice literature, spaced acquisition can afford the learner more varied contextual cues to draw from, thereby facilitating retrieval (Kim et al., 2019). The idea is that acquired information remains vulnerable until it is consolidated, and that context, cues, and repeated exposure can facilitate retention. When content is interleaved, learners recall information from different categories in a mixed order (Foster et al., 2019). In math instruction, for example, this would entail interspersing problems of fraction addition with problems of fraction subtraction, instead of presenting the problems in separate blocks of addition followed by subtraction problems (blocked practice). This staggering of concepts encourages comparing and contrasting, but also resists automaticity by requiring the learner to differentiate processes. Roediger et al. (2019) discuss how spacing and interleaving follow the principle of “practice like the real thing.” Using a baseball analogy, they discuss the importance for a batter to practice spacing because they will never repeatedly receive the same pitch. Additionally, a batter must practice interleaving because they never know what pitch will come next. Distributed practice is essential for effective foundational learning.

Using Bloom’s (1956) taxonomy, higher order learning is built on foundational skills. Once fundamental skills are sufficiently learned and applied, students are prepared to move to more abstract competencies. As learners progress along the novice to expert continuum, they also move from concrete to abstract learning. While learners can be successful workers with only fundamental skills, we argue that abstract thinking is a crucial skill for creativity, critical thinking, and adaptability to a changing work environment. We discuss two techniques to achieve competencies in abstract thinking: mental simulation and reflective meaning making.

#### Mental Simulation and Reflective Meaning Making

Continuing with Roessger’s (2016) novice to expert framework, he summarizes the research on mental simulation as a higher order practice for learners. Mental simulation is the act of mentally previewing scenarios and judging the consequences of actions prior to performing an action. Roessger states the research on mental previewing exclusively proves to be effective for learners who demonstrate domain expertise, suggesting that fundamental skills are prerequisite. Mental simulation is an important technique for learning, as it is more efficient than trial and error, and in some situations less dangerous.

Mental simulation is particularly effective when experts are confronted with unexpected scenarios. Roessger (2016) notes a study in which expert chess players were asked to think aloud about their moves. In each case, the players mentally previewed possible scenarios and predicted potential consequences of their moves several plays into the future. Similarly, an industrial electrician must rely on acute mental simulation prior to repairing a machine, or the consequences could be disastrous, if not deadly. Expert engineers practice mental simulation prior to product design, imagining the product’s mechanics and weight distribution, as well as potential operational failure scenarios. In discussing expert decision making, Roessger (2016, p. 9) writes the following:

In practice, experts must often act with little time to compare alternative courses of action. In such cases, they first identify a familiar environmental cue, then select an action that has previously worked in the presence of that cue (Klein, 1993). The expert then assesses the action’s appropriateness, mentally simulates its implications and, if seemingly effective, acts. In most time-sensitive situations, professionals do not choose from competing actions; they determine a single course of action and modify or reject it only when imagining a flaw in its execution (Klein and Klinger, 1991).

Mental previewing actively engages learners in problem-solving, identifying potential solutions and issues. However, as Roessger emphasizes, this strategy must follow established foundational, domain knowledge.

Mental simulation is a powerful technique for domain experts, but reflective meaning making has the potential to transcend domain knowledge, enabling creativity, transformation, and broad learning transfer. Reflective practice is core pedagogy for many adult educators. However, reflective practice often lacks definitional clarity (Clarà, 2015; Roessger, 2017), and some suggest educators misuse reflective practices entirely (Michelson, 1996, 2011). Based on RFT, Roessger (2017) argues reflection is a functional meaning making process. He defines reflection as “relating an event in a relational network containing the self to an event in an analogous network, under the antecedent control of either incongruous meaning or a request to reconsider meaning” (p. 88). Based on this definition, Roessger (2019) defines three functional methods of meaning making: meaning making, meaning transformation, and reflective meaning transformation. A person engages in *meaning making* when they relate new experiences or concepts to their prior knowledge. For example, a skilled electrician learning the computer programming language, Python, may relate the new programming concepts to their knowledge of programming PLCs. *Meaning transformation* is when a person creates a relationship between two familiar concepts, therefore, transforming the meaning of both concepts. After learning Python, the skilled electrician may form a relationship between the troubleshooting procedures of debugging a computer program and diagnosing a circuitry issue. By doing this, the electrician perceives both troubleshooting issues within a different context. Finally, *reflective meaning transformation* is when a person relates a familiar concept to their identity or sense of self, thus transforming the familiar content as well as transforming themselves. The electrician may find they enjoy programming more than electrical work, prompting a career change and an emerging identity as a programmer.

Mental simulation and reflective meaning making have the potential to further develop skills that are typically highly desirable such as problem-solving and critical thinking. Deliberate practice alone can contribute to a worker’s metacognitive abilities—the ability to self-evaluate and learning to learn. However, these expert techniques can contribute to a worker’s adaptability to change by influencing their efficiency in learning and their ability to transfer their learning to other scenarios.

#### Achieving Efficiency and Transfer

We argue learning efficiency and learning transfer are crucial outcomes for continuous VET. *Learning efficiency* is the balance between learning quickly and learning effectively, while *learning transfer* is the ability to transfer learning from one medium to another. A common criticism of mastery learning is that it is often slower than traditional learning approaches (Slavin, 1987; Kulik et al., 1990; Siddaiah-Subramanya et al., 2017). This is the case for group-paced mastery learning, in which learners in one class or cohort progress through the material based on a “no student left behind” philosophy. However, in recent years, technological progress has made it easier for instructional designers and trainers to personalize learning in a self-paced environment. Additionally, using evidence-based instruction such as deliberate and distributed practice with feedback efficiently solidifies learning. While repeated study may make students feel as though they are learning faster, it creates an illusion whereby students become confident although they have learned significantly less (Karpicke and Grimaldi, 2012; Roediger et al., 2019). Ultimately, however, deliberate, repeated practice improves metacognition, allowing a student to properly self-evaluate and increase efficiency as they progress on the novice to expert continuum.

Learning transfer has been crucial in VET for over a century, with studies examining transfer in various industries and skills (Baldwin and Ford, 1988). Studies on transfer continue, with debate on whether transfer can occur outside of a domain (Daley, 2001), if transfer can occur at all (Detterman and Sternberg, 1993), or, if it does occur, to what extent? Since Baldwin and Ford’s (1988) meta-analysis on the transfer of skills, additional research (see Ford and Weissbein, 1997; Burke and Hutchins, 2007; Blume et al., 2010) has asked three guiding questions:

1. What learner characteristics influence transfer?
2. What instructional or task design factors influence transfer?
3. What environmental or workplace factors influence transfer?

In a recent review, Tonhäuser and Büker (2016) found that at the organizational level, social support—support and feedback by supervisors and peers—had strong evidence for transfer outcomes, as well as a climate that encouraged practicing learned skills in different contexts. For individual characteristics, they found positive relationships between transfer and motivation, self-efficacy, conscientiousness, and openness to experience. Adverse affective traits—neuroticism, anxiety, and emotional instability—had a negative relationship to transfer outcomes. For training interventions, the relevance of the activity, setting learning goals, behavioral modeling, and feedback led to positive learning transfer outcomes.

While there is some evidence that deliberate practice influences near transfer—transferring learning from one activity to a similar activity (Butler, 2010; Butler et al., 2017; Agarwal, 2019), learners likely need to engage in mental simulation or reflective meaning making to make larger contextual leaps. To achieve far or lateral transfer—transfer that extends tasks and domains, educators and trainers must assist learners in making meaning of the content. Roumell (2019) calls this “mindful transfer” and suggests strategies such as examining personal commitments, crafting stories, and reflecting on and developing personal ownership of newly acquired content. This aligns with the current transfer research reflected in Tonhäuser and Büker (2016). Within the functional contextualist approach to mastery learning, these strategies reinforce contextualized, reflective meaning making. In sum, to achieve effective transfer outcomes, learners should relate the material to prior experience, to their historical and sociocultural context, and to themselves.

## Summary and Conclusion

In this article, we argued a functional contextualist approach to mastery learning is an effective method for learners to achieve higher order learning outcomes. We situate functional contextualism within the broader VET literature, and within mastery learning, we posit that functional contextualism extends mastery learning beyond its procedural roots. We presented research from cognitive and behavioral science addressing pedagogical techniques for teaching learners on the novice to expert continuum. Additionally, we proffer mental simulation and reflective meaning making as suitable methods to build upon foundational domain knowledge and achieve efficiency and transfer outcomes.

While comprehensive and systemic policy changes are likely the best solution for addressing the continuous and disruptive nature of VET, a functional contextualist approach to mastery learning which recognizes the procedural development of skills and encourages higher order thinking can provide learning theory and pedagogical direction to educators, trainers, and learners. Basic skill development may seem purely instrumental and non-critical, but it is necessary and foundational for higher order thinking and meaning making. Finally, this contextualized approach to learning contributes to the ongoing development of life-literacies, adaptability, learning dispositions, agency freedom, and work capabilities that serve adult learners across multiple life domains, ultimately improving our broader “vocation of being human.”

## Author Contributions

All authors listed have made a substantial, direct and intellectual contribution to the work, and approved it for publication.

## Conflict of Interest

The authors declare that the research was conducted in the absence of any commercial or financial relationships that could be construed as a potential conflict of interest.
